# Perioperative management of surgical orthodontic treatment in a patient with glucose transporter 1 deficiency: report of a case and review of the literature

**DOI:** 10.1186/s13741-022-00287-8

**Published:** 2022-12-16

**Authors:** Nishiyama Kyoko, Hamada Masakazu, Nabatame Shin, Shimizu Hidetaka, Uzawa Narikazu

**Affiliations:** 1 Department of Oral and Maxillofacial Surgery II, Graduate School of Dentistry, Osaka University, 1-8 Yamadaoka, Suita, Osaka, 565-0871 Japan; 2 Department of Pediatrics, Graduate School of Medicine, Osaka University, 2-2 Yamadaoka, Suita, Osaka, 565-0871 Japan; 3 Department of Oral and Maxillofacial Surgery, Saiseikai Suita Hospital, 1-2 Kawazonocho, Suita, Osaka, 564-0013 Japan

**Keywords:** Glucose transporter type 1 (GLUT1) deficiency, Jaw deformity, Ketogenic diet, Orthognathic surgery, Perioperative management

## Abstract

**Introduction:**

Glucose transporter 1 (GLUT1) deficiency is a rare cerebral metabolic disorder caused by the shortage of glucose supply to the brain. For this disease, ketogenic diet therapy is essential. In addition, perioperative management requires not only the continuation of ketogenic diet therapy but also the management of nausea/vomiting, diarrhea, seizures, and infection. However, there have been few reports regarding oral and maxillofacial surgery.

**Case presentation:**

We describe a patient with GLUT1 deficiency who underwent orthognathic surgery. An 18-year-old man was referred to our hospital with the chief complaint of mandibular regression. Surgical tolerance was assessed by a fasting test and tooth extraction under general anesthesia, and orthognathic surgery was then performed. For orthognathic surgery, the mandibular dentition had scissor-like occlusion, and it was difficult to arrange the mandible. Therefore, we decided to perform maxillary osteotomy first. After the mandibular dentition was arranged by maxillary osteotomy, sagittal split ramus osteotomy (SSRO) was performed. Intermaxillary fixation (IMF) was necessary for SSRO, and caution was needed to prevent suffocation. The orthognathic surgery was successful, although complications, such as vomiting, diarrhea, and seizures, developed.

**Conclusion:**

Surgical orthodontic treatment in GLUT1 deficiency can be performed relatively safely by maintaining the diet, taking measures against epilepsy and vomiting, and using antimicrobial agents in close collaboration with pediatricians, anesthesiologists, pharmacists, and nutritionists.

## Background

Glucose transporter 1 (GLUT1) deficiency is a rare type of metabolic encephalopathy caused by mutations in the coding gene for GLUT1 (*SLC2A1*), which was first reported by De Vivo et al. in 1991 (De Vivo et al. 1991). It is caused by the failure of the central nervous system to take up glucose, a substrate of energy metabolism in the brain (Ito et al. 2015; Larsen et al. 2015; Leen et al. 2010; Tang et al. 2017). For this disease, a ketogenic diet is used to provide ketone bodies to supplement the energy needs of the brain with sources other than glucose (Gumus et al. 2015; Klepper 2008).

More than 200 cases have been reported, mainly in Europe and the USA, and 57 cases were confirmed in a nationwide survey in Japan in 2011 (Ito et al. 2015; Klepper 2012). Perioperative management of GLUT1 deficiency, including oral surgery, has rarely been reported (Motoki et al. 2020). This report describes the perioperative management of surgical orthodontic treatment in a patient with GLUT1 deficiency under ketogenic diet therapy. To our knowledge, this is the first report of treatment of a patient with GLUT1 deficiency who underwent surgical orthodontic treatment under ketogenic diet therapy.

## Case presentation

**Fig. 1:**
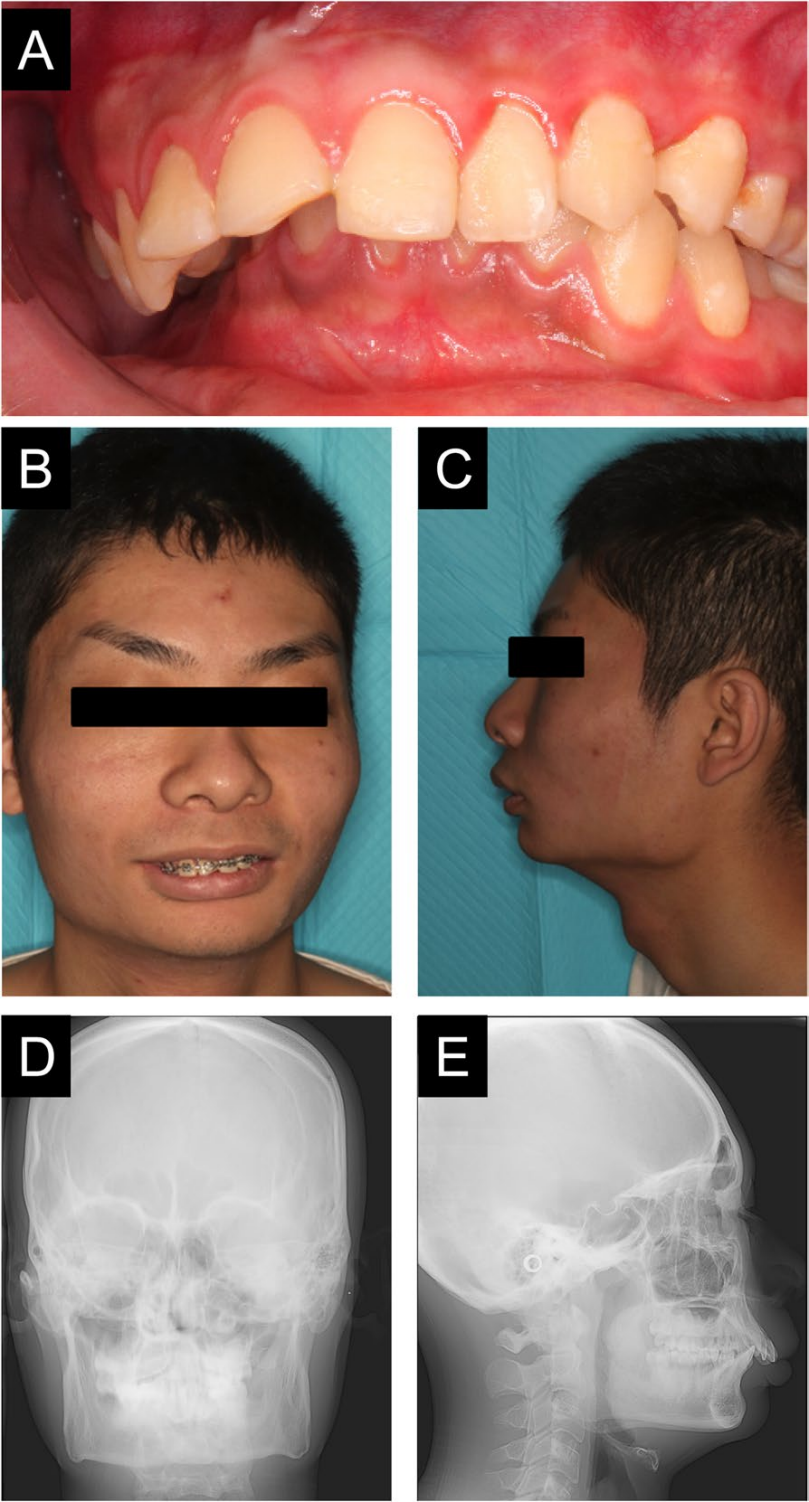
Preoperative photographs and radiographs. Intraoral photographs at the time of tooth extraction (**A**). Frontal view (**B**) and lateral view (**C**) on facial photographs before maxillary osteotomy. Frontal cephalogram (**D**) and lateral cephalogram (**E**) showing radiographic findings at the time of tooth extraction

An 18-year-old man with GLUT1 deficiency was referred to the Department of Oral and Maxillofacial Surgery at Osaka University Dental Hospital with the chief complaint of mandibular regression. He had epilepsy, intellectual disability, cerebellar ataxia, spastic paraplegia, and other symptoms associated with GLUT1 deficiency and was undergoing ketogenic diet therapy. Habitual epilepsy, such as tonic-clonic convulsion of the face and extremities followed by frequent vomiting, was observed several times a year. There was overjet of 7 mm and overbite of 4 mm, indicating an overbite. The mandibular anterior teeth were in contact with the palate, and the mandible receded (Fig. 1A). His facial appearance was asymmetrical from the frontal view and convex from the lateral view, and retraction of the mantle was observed (Fig. 1B–E). He was diagnosed with skeletal mandibular recession, and surgical orthodontic treatment was indicated. As it was unclear whether the patient was able to tolerate surgery, he was evaluated for surgical tolerance by a fasting test and tooth extraction under general anesthesia, and orthognathic surgery was then performed (Table 1). We previously reported this patient with GLUT1 deficiency who underwent tooth extraction under general anesthesia (Motoki et al. 2020). Based on the experience of tooth extraction under fasting and general anesthesia, surgical orthodontic treatment was judged as possible.

**Table 1:**
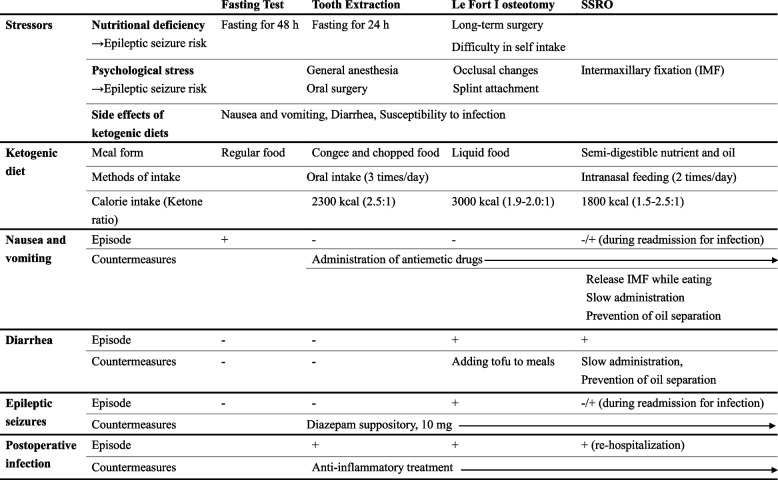
Perioperative management plan, side effects, and countermeasures

Osteotomy of the maxilla and mandible was required. However, as the mandibular dentition was difficult to align due to the scissor-like occlusion, we decided to precede with osteotomy of the maxilla (4⏌, ⎿4 extractions, 3-segment Le Fort I) and perform sagittal split ramus osteotomy (SSRO) after the mandibular dentition was aligned. The patient underwent maxillary osteotomy at the age of 22 years. Nutritional management was performed by a ketogenic diet during hospitalization. Metoclopramide, an antiemetic agent, was administered before meals as during tooth extraction no nausea or vomiting developed. However, diarrhea was frequent and continued for approximately 1 week. Diarrhea was observed up to 9 times per day. Stool culture was performed, but it was negative for pathogenic bacteria. For frequent diarrhea, we increased the protein intake by adding tofu, which contains few carbohydrates, but the symptom did not improve. On the 3rd day of surgery, a mild epileptic seizure was observed, and a 10-mg diazepam suppository was inserted, and improvement was observed. As the plastic syringe used for meals dissolved due to the medium chain triglycerides (MCT) oil used in the ketogenic diet, a glass syringe was used instead. The patient was discharged with preventive administration of clarithromycin because the CRP value was 1.9 mg/dL, although there was no evidence of bacterial infection. After discharge from the hospital, transient alopecia areata, probably due to stress, was observed during follow-up after maxillary osteotomy.

**Fig. 2:**
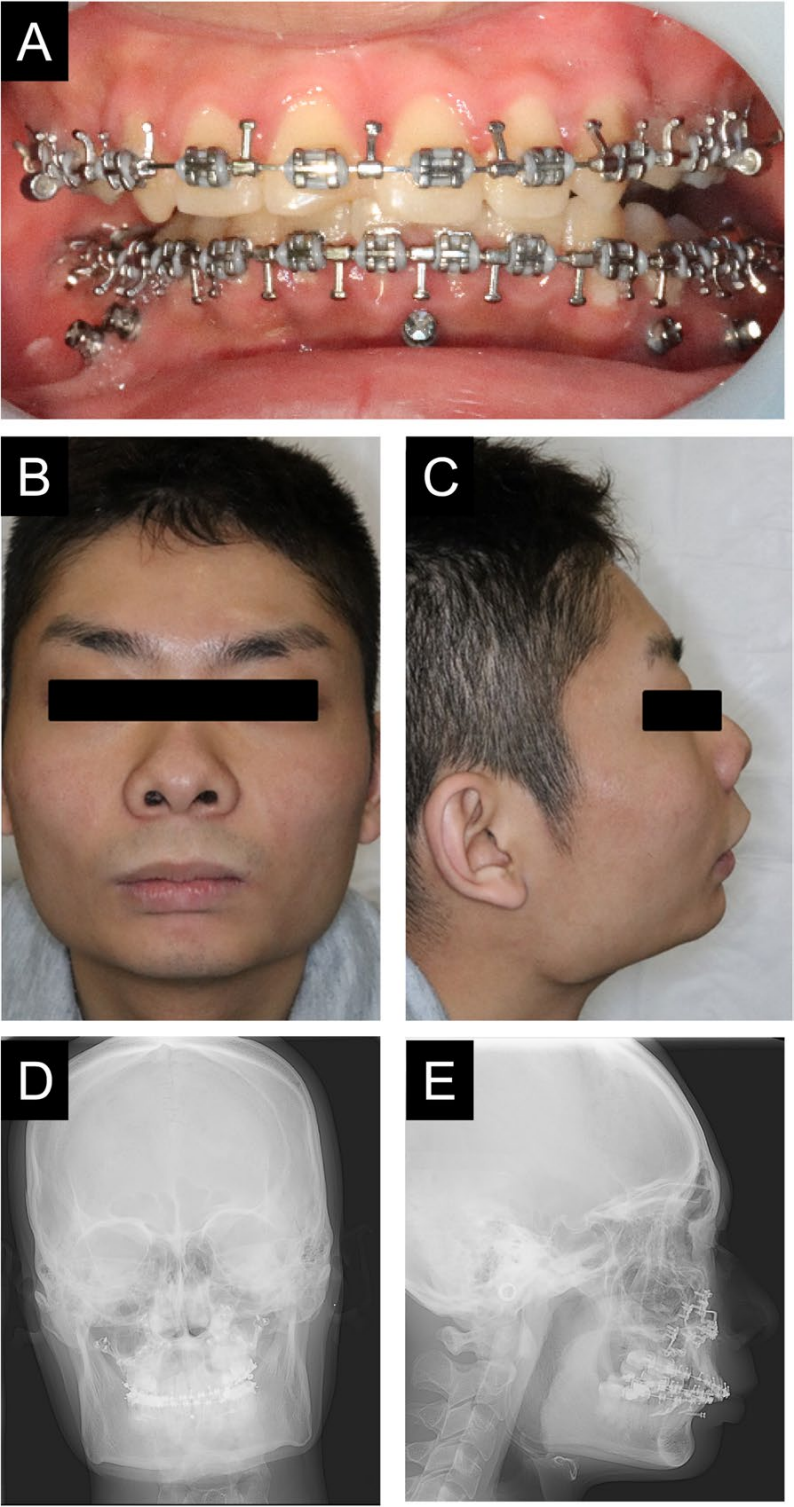
During treatment photographs and radiographs. Intraoral photographs before mandibular osteotomy (**A**). Frontal view (**B**) and lateral view (**C**) on facial photographs before mandibular osteotomy. Frontal cephalogram (**D**) and lateral cephalogram (**E**) showing radiographic findings before mandibular osteotomy

**Fig. 3:**
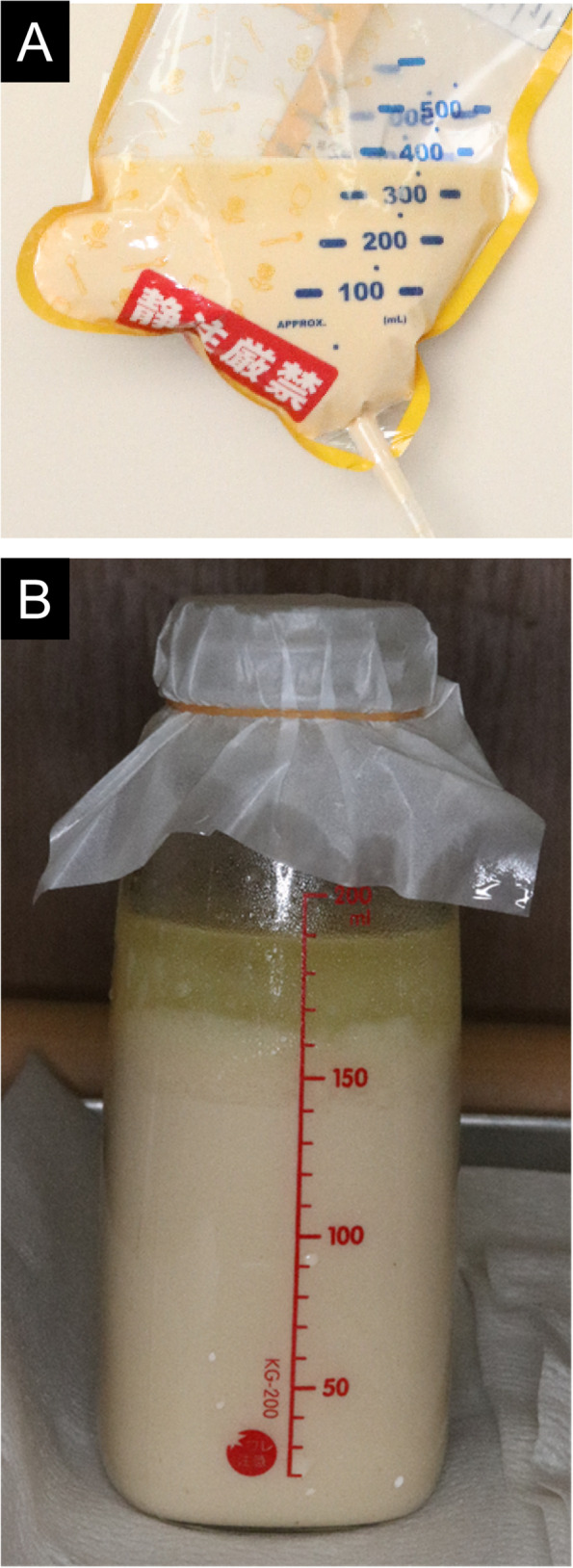
Tube feeding for ketogenic diet therapy. A tube feeding package for ketogenic diet therapy (**A**). Oil separation image (**B**)

**Fig. 4:**
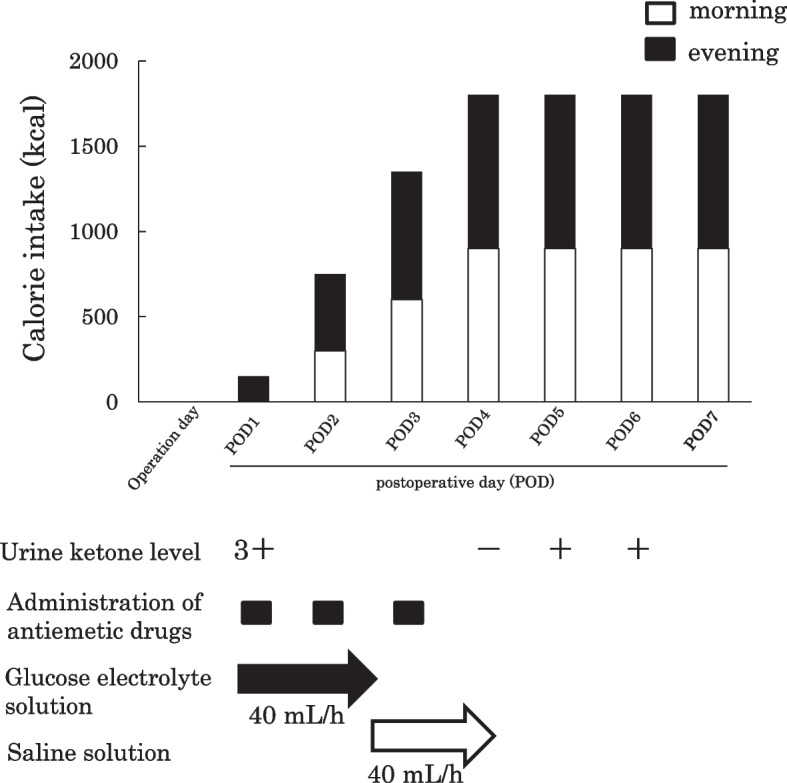
Postoperative ketogenic diet schedule. Meals were increased by 150 kcal per meal, and on the fourth postoperative day, 900 kcal per meal, or 1800 kcal per day, was achieved

**Fig. 5:**
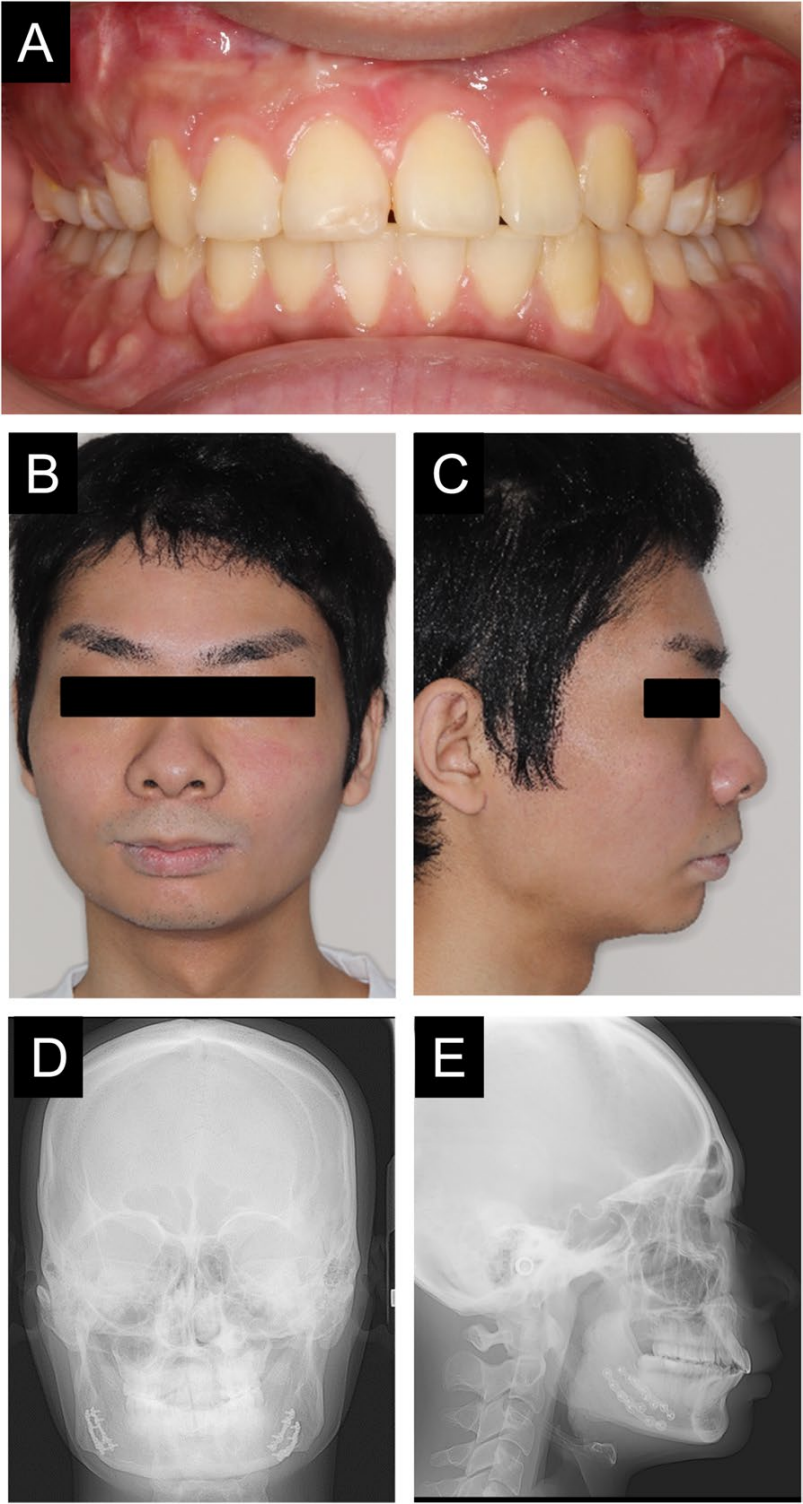
Postoperative photographs and radiographs. Intraoral photographs after orthognathic surgery (**A**). Frontal view (**B**) and lateral view (**C**) on facial photographs after orthognathic surgery. Frontal cephalogram (**D**) and lateral cephalogram (**E**) showing radiographic findings after orthognathic surgery

After the mandibular alignment was completed (Fig. 2A–E), SSRO was performed at the age of 23 years. The problems of perioperative management were the need for intermaxillary fixation (IMF), countermeasures against diarrhea, and reduction of the burden on the family. After the previous operation, maxillary osteotomy, the biggest burden for the patient and family was food intake and care. Therefore, after consultation with the patient’s family and the pediatrician, it was decided that the patient would receive tube feeding instead of oral feeding after surgery (Fig. 3A). As a countermeasure for gastrointestinal symptoms, the infusion rate was slowed down to approximately 1 h. The ketone ratio was adjusted by mixing 300 mL of Glucerna with 66 mL of olive oil, instead of MCT oil because MCT oil, which was used previously, dissolved the plastic injection device. As the oil separated during injection (Fig. 3B), resulting in poor dripping, heating the mixture to approximately 40 °C beforehand enabled relatively good injection. Vomiting was observed during the previous fasting test; therefore, IMF was released during the injection and 1 h after the injection in order to open the mouth to prevent aspiration and choking associated with vomiting. After fasting under general anesthesia, the infusion volume was started at a small amount and gradually increased to allow the patient to become accustomed to the ketogenic diet (Fig. 4). As a result of these measures, there was almost no vomiting or diarrhea after the resumption of the ketogenic diet. However, infection was observed on the 10th day of surgery, which was resolved by intravenous sulbactam sodium, and the patient was discharged. Two weeks after discharge from the hospital, another infection was observed, and anti-inflammatory treatment was necessary. Currently, approximately 1 year has passed since the surgery, but no recession has been observed, and good occlusal condition was confirmed (Fig. 5A–E).

## Discussion and conclusions

GLUT1 deficiency is caused by a lack of energy in the brain due to the inability of the central nervous system to take up glucose, a substrate of brain energy metabolism, resulting in symptoms such as intellectual disability, intractable epilepsy that does not respond to conventional antiepileptic drug therapy, spastic paralysis, cerebellar ataxia, and involuntary movements such as dystonia, myoclonus, or exertion-induced dyskinesia (Ito et al. 2015; Larsen et al. 2015; Leen et al. 2010; Tang et al. 2017). For this disease, a ketogenic diet is used to provide ketone bodies to supplement the energy needs of the brain with sources other than glucose (Gumus et al. 2015; Klepper 2008). A ketogenic diet should be started as soon as the diagnosis is made because it significantly improves the patient’s quality of life if started before the brain damage progresses (Klepper et al. 2020; Veggiotti and De Giorgis 2014). In the present case, the patient was started on a ketogenic diet immediately after diagnosis; the symptoms of our patient significantly improved, although he still had epilepsy, spastic paralysis, and cerebellar ataxia. In addition, epilepsy, ataxia, and involuntary movements are known to be aggravated during fasting and fatigue (Hao et al. 2017; Schwantje et al. 2020), and epilepsy due to surgical invasion during surgical correction treatment was of concern. A single mild epileptic seizure was noted during the first orthognathic surgery, maxillary osteotomy, but this was not different from the normal frequency. During the second orthognathic surgery, SSRO, postoperative infection developed, and the frequency of epilepsy (tonic, atonic, and convulsive seizures) increased transiently. This was thought to be due to excessive stress and poor ketogenic diet intake.

In order to monitor ketogenic diet, serum ketone and urine ketone are utilized (Kossoff et al. 2018). Although serum ketone is more accurate, it is more expensive and requires finger pricks (Kossoff et al. 2018). In the present case, serum beta-hydroxybutyrate (BOH) was not always available for measurement in our hospital. Therefore, serum BOH was monitored only preoperatively and intraoperatively as a special case. After the surgery, we monitored urine ketones.

Patients with GLUT1 deficiency, such as the present patient, often have blunted pain senses. This makes it difficult to recognize the signs of infection and the degree of improvement. In the first surgery reported by Motoki et al., post-extraction infection developed, but improvement was observed after administration of antimicrobial agents and cleaning of the extraction socket (Motoki et al. 2020). However, due to pain insensitivity, the patient did not complain of pain at an early stage, and infection was only found when the inflammation was considerably advanced. After maxillary osteotomy, there was no notable swelling at the time of discharge, but the CRP was 1.9, which was abnormal; therefore, the patient was discharged under continuous clarithromycin administration. However, no abnormalities were observed in the healing process of the wound after discharge. During the last SSRO, infection was observed on the 10th day of surgery. However, 2 weeks after discharge, infection was again observed, and anti-inflammatory treatment was required. The patient did not complain of symptoms until pain and swelling became marked. This case is a reminder of how important the signs of pain are for clinicians.

Many adverse effects have been reported to develop during ketogenic diet therapy. Side effects of ketogenic therapy include gastrointestinal disorders, hyperlipidemia, coronary artery disease, renal calculi, slowed growth, cardiac abnormalities, pancreatitis, and hepatic dysfunction (Kossoff et al. 2018). As for gastrointestinal symptoms, constipation is very common, but not diarrhea (Kossoff et al. 2018). MCT is known to cause gastrointestinal side effects such as diarrhea (Liu 2008). In the present case, no diarrhea was observed during the fasting test or tooth extraction. However, diarrhea occurred frequently after the more invasive maxillary osteotomy. This diarrhea may be caused by several factors, including the change in the food form, increase of MCT intake, and an antibiotic-induced bacterial shift phenomenon. Although the patient’s ketogenic diet was usually solid food, it was changed to a completely liquid formula containing a large amount of MCT. For intractable diarrhea, we increased the amount of protein and dietary fiber in the diet, which contained few carbohydrates, for digestibility and nutritional support, but the symptom did not improve immediately. It was considered that diarrhea improved slowly because the diet consisted mainly of fat.

Furthermore, Kossoff et al. (2018) reported that vomiting and abdominal pain are the common side effects. In the present case, similar to that reported by Motoki et al., the patient vomited during the fasting test, and the antiemetic drug metoclopramide was administered before resuming eating (Motoki et al. 2020). In the case of tooth extraction and maxillary osteotomy, administration of antiemetic metoclopramide before the start of meals prevented vomiting. In the postoperative period of mandibular osteotomy, IMF is required, and vomiting during IMF may lead to choking. In the present case, postoperative management by spontaneous oral intake, such as ensuring sufficient food intake and IMF time, was considered difficult. Therefore, we decided to manage the patient by tube feeding, which makes it easy to control the calorie intake and meal time, and we released IMF for an hour after the formula infusion to reduce the risk of aspiration of gastric contents. In addition, the amount of food was gradually increased in order for the patient to gradually become accustomed to the ketogenic diet (Fig. 4). The patient’s family was also instructed to give gummies from early on to prevent vomiting during IMF. Although no vomiting was observed during or immediately after meals, it was observed when the patient went to the toilet at night on the 12th day of the surgery, but aspiration and choking were avoided because IMF was quickly released. As IMF is necessary for surgical orthodontic treatment, it was considered important to take measures.

In summary, we reported the surgical orthodontic treatment of a patient with GLUT1 deficiency, a rare disease. Surgical orthodontic treatment in GLUT1 deficiency can be performed relatively safely by maintaining the diet, taking measures against epilepsy and vomiting, and using antimicrobial agents in close collaboration with pediatricians, anesthesiologists, pharmacists, and nutritionists.

## Data Availability

The raw data supporting the conclusions of this article will be made available by the authors, without undue reservation.

## Abbreviations

GLUT1: Glucose transporter 1
SSRO: Sagittal split ramus osteotomy
MCT oil: Medium chain triglycerides oil
IMF: Intermaxillary fixation
